# Supplementary material for: Single and Mixed Strains of Probiotics Reduced Hepatic Fat Accumulation and Inflammation and Altered Gut Microbiome in a Nonalcoholic Steatohepatitis Rat Model
Source: Biomedicines. 2024 Aug 14;12(8):1847. doi: 10.3390/biomedicines12081847 (PMC11605219; doi:10.3390/biomedicines12081847)

Sample size calculation

The number of rats in this study was calculated from the results of Werawatganon (1) using the G Power program**.** The authors studied the effect of *L. plantarum* on hepatic malondialdehyde (MDA) levels in a NASH model. The results in each group were as follows: control (n=8), 6.48±4.03 nmol/mg of tissue; NASH (n=8), 12.41 ± 7.98 nmol/mg of tissue; and NASH+*L. plantarum* (n=8), 1.66 ± 0.19 nmol/mg tissue (data were presented as mean ± SEM). SD was then calculated from SEM. The mean and SD of MDA levels in each group were used to calculate the sample size using G*Power 3.1.9.2. The total sample size was 28; therefore, the number of rats per group was 7.

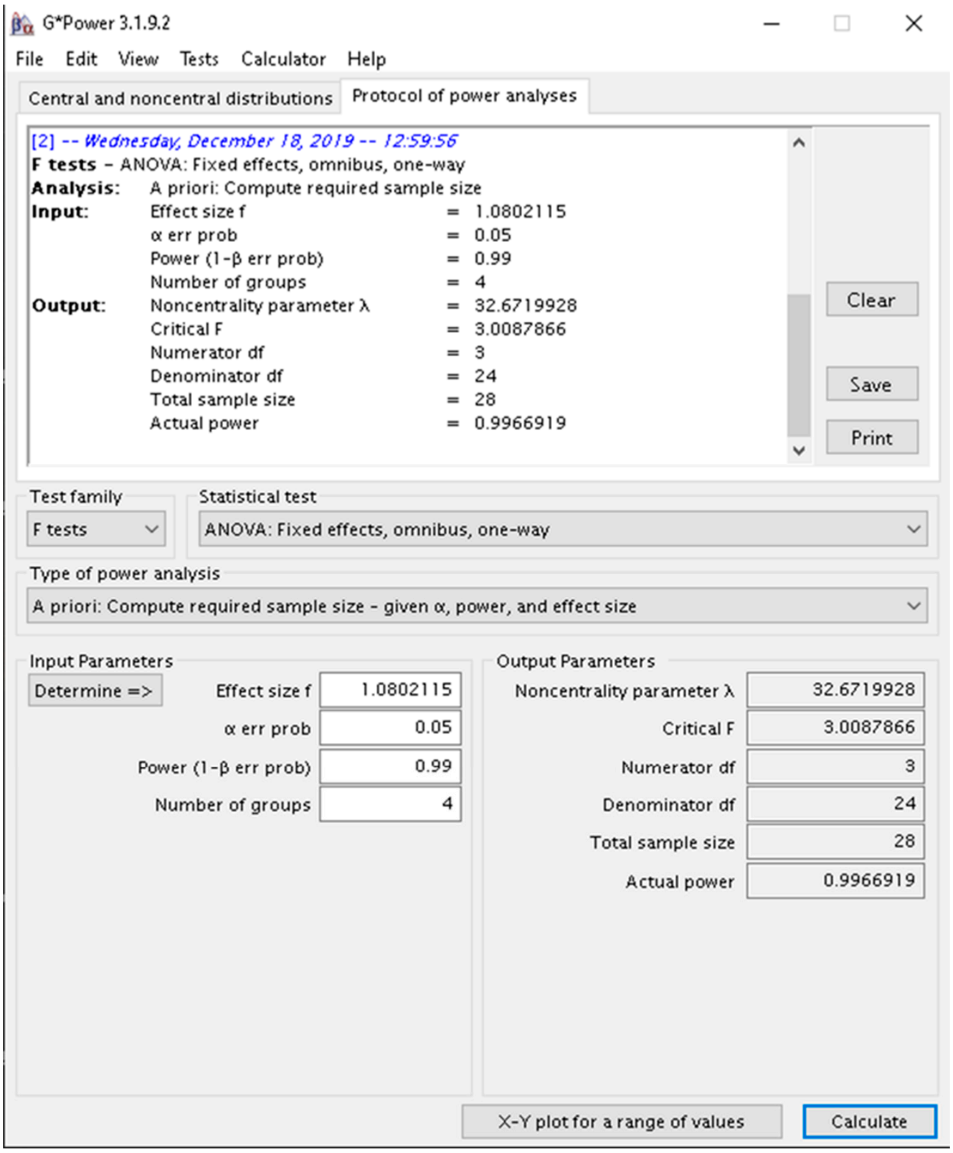

Sample size calculation by G power program

1. Werawatganon D, Somanawat K, Tumwasorn S, Klaikeaw N, Siriviriyakul P. Lactobacillus plantarum attenuates oxidative stress and liver injury in rats with nonalcoholic steatohepatitis. Pharmacognosy Magazine. 2018;14(58):471-6.

Supplementary Figure S1: Beta diversity between control and NASH groups.

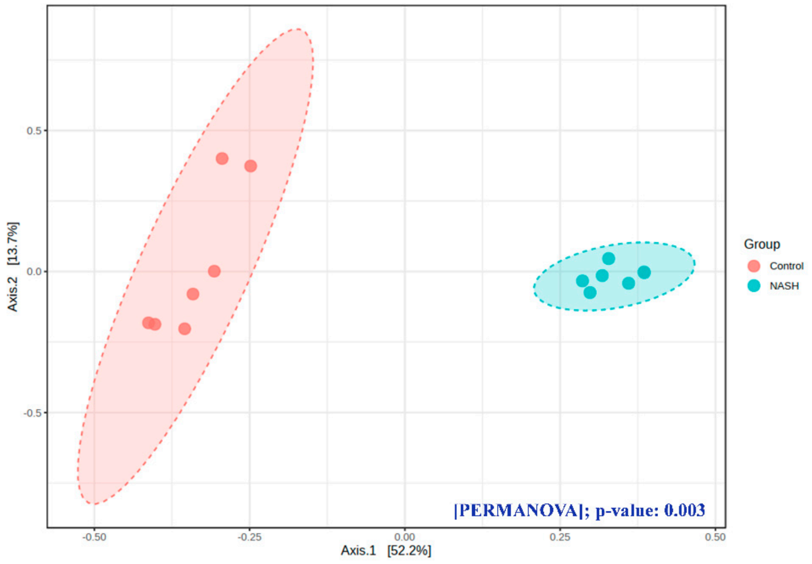

Supplementary Figure S2: Beta diversity between control and single-strain groups.

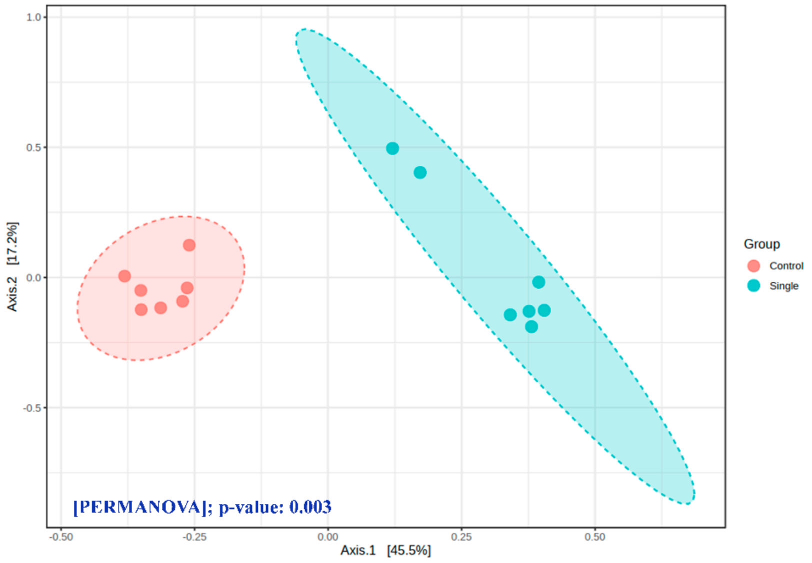

Supplementary Figure S3: Beta diversity between control and mixed-strain groups.

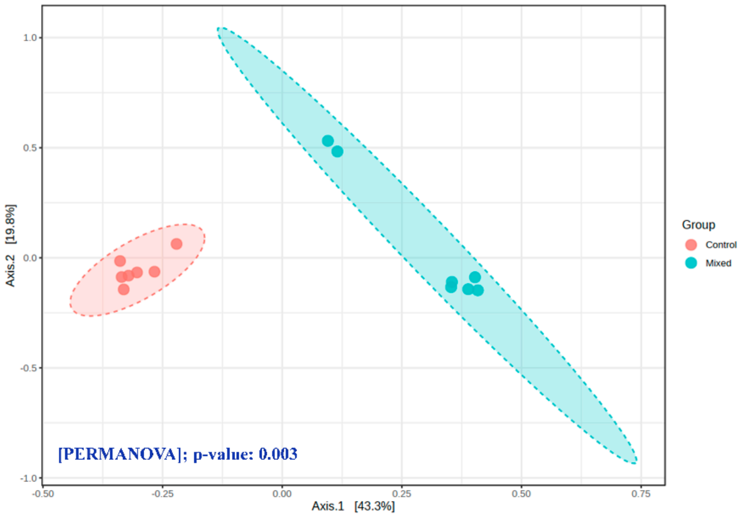

Supplementary Figure S4: Gut microbial composition between control and NASH groups.

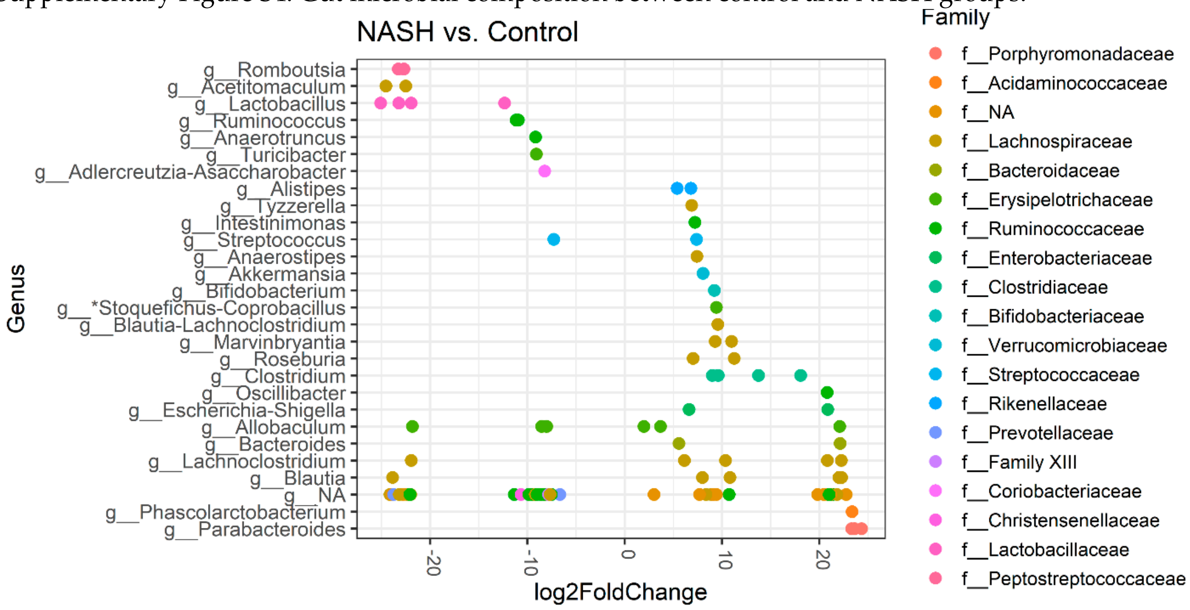

Supplementary Figure S5: Gut microbial composition between NASH and single-strain groups.

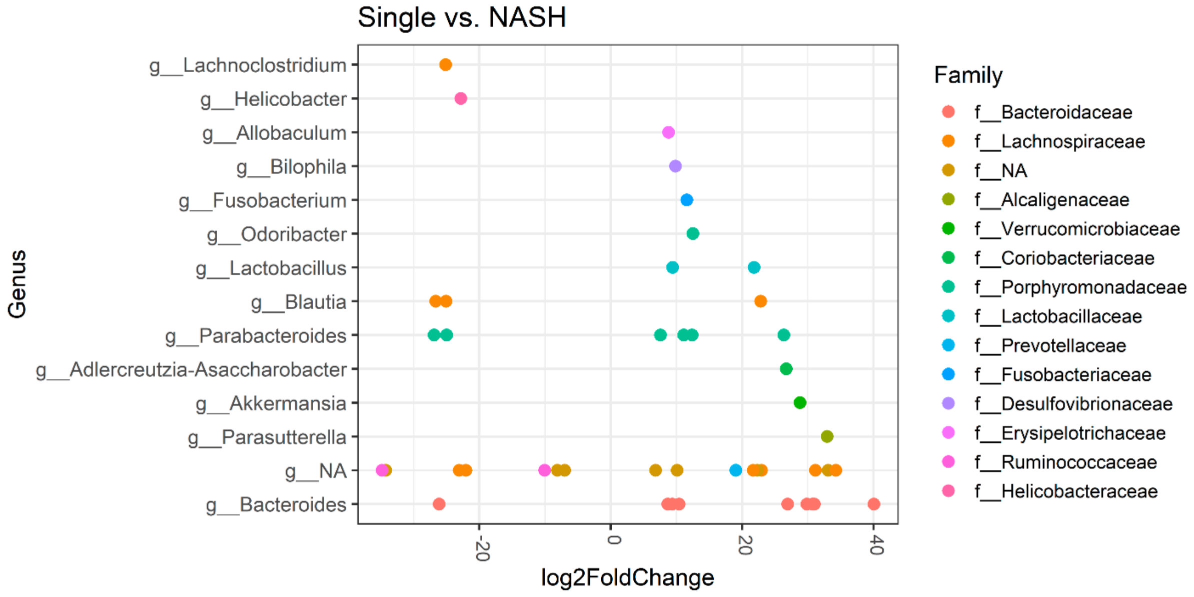

Supplementary Figure S6: Gut microbial composition between NASH and mixed-strain groups.

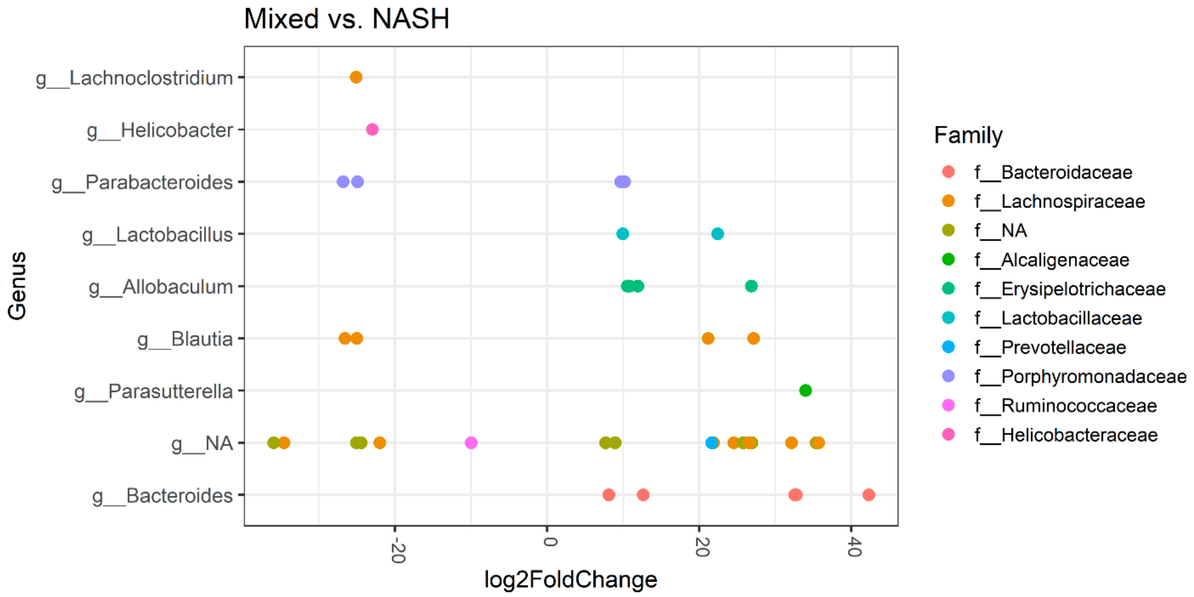

Supplement: Supplementary file 1 [file biomedicines-12-01847-s001.zip › biomedicines-3113055-supplementary.pdf]
